# Identification of the interaction between MAPK1 and *Eimeria acervulina* serine protease inhibitor: a preliminary functional study: Interaction between MAPK1 and *Eimeria acervulina* serine protease inhibitor

**DOI:** 10.3724/abbs.2024095

**Published:** 2024-06-28

**Authors:** Liyin Lian, He Sun, Jing Wang, Wanjing Li, Yifan Sheng, Xinyue Gong, Qian Sun, Pu Wang, Yadong Zheng, Houhui Song

**Affiliations:** College of Animal Science and Technology & College of Veterinary Medicine Zhejiang A&F University Key Laboratory of Applied Technology on Green Eco-Healthy Animal Husbandry of Zhejiang Province Provincial Engineering Research Center for Animal Health Diagnostics & Advanced Technology Zhejiang International Science and Technology Cooperation Base for Veterinary Medicine and Health Management China Australia Joint Laboratory for Animal Health Big Data Analytics Hangzhou 311300 China

*Eimeria spp*. can invade different intestines of chickens. Among them, *Eimeria acervulina* ( *E*. *acervulina*, *Ea*) is the most virulent, and it is characterized by disruption of the intestinal nutrient uptake mechanism, leading to weight loss and even death. As a result, coccidia have caused a heavy burden on the poultry industry [1]. Currently, understanding the invasion mechanism of *E*. *acervulina* in host cells is the basis for developing the most effective preventive method for coccidiosis. However, the specific mechanism of *E*. *acervulina* invasion is unclear, so the interactions between parasite and host cells need to be studied in depth.

Serine thiol proteinase inhibitors widely exist in bacteria, viruses and parasites. It can protect against the dissolution of host proteins and the development of pathogens in the process of pathogen invasion [2]. The serine protease inhibitor (SERPIN) of *Toxoplasma gondii*, which has the ability to inhibit trypsin activity, was the first SERPIN to be studied in parasites. SERPIN in *T*. *gondii* may play an important role in protecting against the degradation of host intestinal proteases and reducing the ability of host proteins to hydrolyse *T*. *gondii* [3]. SERPIN in *T*. *gondii* can also promote the growth of tachyzoites in the host. Subsequently, a 79-amino acid protease inhibitor, SERPIN, which can completely inhibit the activity of bacterial subtilisin, was found in *Neospora caninum* [4]. SERPIN plays an important role not only in parasite physiology but also in the interactions with the host. Real-time quantitative PCR analysis at all developmental stages of *E*. *tenella* showed that SERPIN1 is highly expressed in sporozoites [5]. Previous studies have shown that the secreted SERPIN protein in sporozoites may also regulate the host immune pathway. In *Schistosoma mansoni*, SERPIN can inhibit neutrophil proteases and regulate the degradation of tissues to promote the migration of parasites in the host [6]. SERPIN secreted by parasites can also be used to protect themselves from degradation by host proteases, thus manipulating the host response to parasites. Studies have shown that they are mainly involved in physiological processes such as blood sucking, digestion, reproduction and immune response and affect the interaction between parasites and hosts [7]. SERPIN from *Trichinella spiralis* (TsSPI) is not directly involved in the growth and reproduction of parasites but regulates the interaction between *T*. *spiralis* and its host to a certain extent. TsSPIs can regulate the polarization of macrophages and subsequently affect the balance among host inflammatory factors to regulate the host immune response and create a favorable environment for the colonization of *Trichinella spiralis* in the host [8]. *Taeniasis solium* SERPIN can play a biological role by participating in the inflammatory and apoptotic pathways of the host [9].

Previous studies have shown that SERPIN plays important roles during host-cell invasion, and 66 related proteins that interact with *E*. *tenella* SERPIN on the host have been preliminarily screened [10]. However, few studies have focused on the binding partner of Ea-SERPIN. To identify the ligand-binding partner molecules that may play an important role in the invasion process of *E*. *acervulina*, a yeast two-hybrid system was used to screen the associated proteins from the yeast complementary DNA (cDNA) library of chicken duodenal epithelium cells using Ea-SERPIN as bait.

In this study, a close genetic relationship was identified between *E*. *acervulina* and *E*. *maxima* and between *E*. *acervulina* and *T*. *gondii*. The protein homology of SERPIN between *E*. *acervulina* and *E*. *maxima* was 87%, and that between *E*. *acervulina* and *Toxoplasma gondii* was 43% ( Supplementary Figure S1). The yeast cDNA library of chicken duodenal epithelium cells in the pGADT7 vector was constructed using a Matchmaker Library Construction and Screening kit (Clontech, Palo Alto, USA) as the prey. Preys containing a Gal4 activation domain (AD prey) were transformed into the yeast strain Y187. The efficiency of transformation and the size of the insert fragment satisfied the quality requirements of the yeast library.

For yeast two-hybrid screening, SERPIN was amplified by polymerase chain reaction (PCR) using the forward primer 5′-CCCCATATGATGGCATTATTAAGTAAATTAACTCG-3′ and the reverse primer 5′-CCCCTGCAGTTACTGCTGTGCAGCTGTCGGGTCAG-3′ from *E*. *acervulina* cDNA and then ligated into the *Nde*I- *Pst*I sites of pGBKT7 as a bait. The recombinant plasmid was transformed into Y2H GOLD yeast cells, and the transformants were separately grown on plates containing minimal yeast medium without tryptophan (SD/‒Trp), SD/‒Trp supplemented with 40 μg/mL X-α-Gal (SD/‒Trp/X) and SD/‒Trp supplemented with 40 μg/mlL X-α-Gal and 125 ng/mL aureobasidin A (SD/‒Trp/X/A). Then, the expression of the recombinant plasmid pGBKT7-SERPIN in the yeast was detected by western blot analysis using an anti-myc monoclonal antibody (Epitomics, Burlingame, USA) ( Supplementary Figure S2). The baits were tested for self-activation, and the results showed that the Ea-SERPIN protein can be expressed in yeast without self-activation ( Figure 1A). Next, we carried out yeast two-hybrid screening using Ea-SERPIN as a bait. To identify the host proteins that interact with Ea-SERPIN, we mated Y2HGold cells harboring the pGBKT7-Ea-SERPIN plasmid with Y187 cells harboring the chicken duodenal epithelium cDNA library. After mating of pGBKT7-Ea-SERPIN-transformed Y2HGold with Y2HGOLD containing the chicken duodenal epithelium cDNA library, we obtained five positive clones in selection medium (SD/‒Leu/‒Trp/X/A). The prey plasmids were rescued through the transformation of *Escherichia coli* DH5α cells. The specific insert on each prey plasmid was amplified by PCR using primers originated from the vector backbone and analyzed by gel electrophoresis (data not shown). To eliminate false positive hits and retest the specificity of interactions, each of the five prey plasmids was tested for self-activation. Prey plasmids without self-activation were cotransformed with pGBKT7-Ea-SERPIN into Y2H Gold cells, and the cotransformants were tested on SD/‒Ade/‒His/‒Leu/‒Trp/X/A plates [2]. The interactions between Ea-SERPIN (200‒400 aa) or Ea-SERPIN (23‒200 aa) and the prey plasmid were separately identified. The results indicated that MAPK1 still had a positive interaction with the SERPIN bait. The host protein MAPK1 (27‒361aa) obviously interacts with SERPIN (200‒400 aa) but not with SERPIN (23‒200 aa) according to *in vitro* yeast two-hybrid assays ( Figure 1B). The results suggested that MAPK1 and SERPIN have more than one interaction region. These results showed that the insert contained the full-length cDNA of mitogen-activated protein kinase 1 (MAPK1), which shares 100% similarity with the known gene of *MAPK1* (XM_015877532). Thus, the host protein MAPK1 was shown to interact with SERPIN and was further analyzed by co-immunoprecipitation. The full-length sequence of SERPI was cloned and inserted into pcDNA3.1-Myc, and the full-length sequence of MAPK1 was cloned and inserted into pcDNA3.1-HA. 293T cells were transfected with the recombinant plasmids pcDNA-Myc-Ea-SERPIN and pcDNA-HA-MAPK1, pcDNA-Myc-MAPK1 only, or pcDNA-HA-Ea-SERPIN only using Lipofectamine 2000 (Invitrogen, Carlsbad, USA) according to the manufacturer’s instructions. Twenty-four hours after transfection, protein extracts were harvested using lysis buffer and then precleared with protein A beads. The precleared extracts were incubated with protein A beads and anti-Myc (Epitomics, Burlingame, USA) antibodies. The beads were washed sequentially with lysis buffer, and the bound proteins were eluted for western blot analysis using anti-Myc and anti-HA antibodies (Proteintech, Chicago, USA) [ 3, 4]. The results showed that Ea-SERPIN bound to MAPK1 but not to the control protein ( Figure 2A).

**Figure 1:**
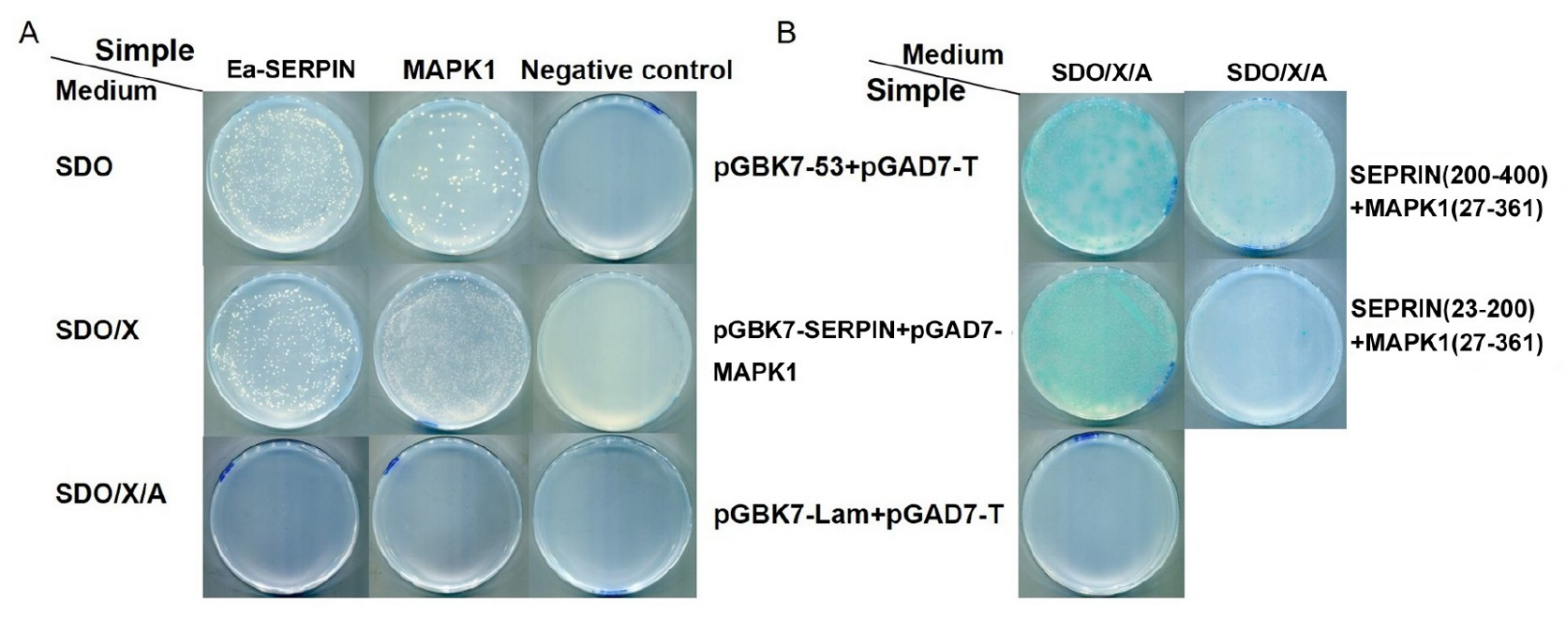
Figure 1 Detection of the SERPIN-MAPK1 protein interaction by yeast two-hybrid assay (A) To evaluate the autoactivation of pGBKT7-SERPIN, the Y187 strains were transformed with pGBKT7-SERPIN. (B) The α-galactosidase activity of the transformants was determined. The combination of plasmids in the Y187 strains is indicated on the right. pGBKT7-53+pGADT7-T and pGBKT7-Lam+pGADT7-T were used as positive and negative controls, respectively. Interaction of SERPIN with MAPK1 in mammalian cells. 293T cells were transfected with the expression plasmid pcDNA-Myc-SERPIN+pcDNA-HA-MAPK1.

**Figure 2:**
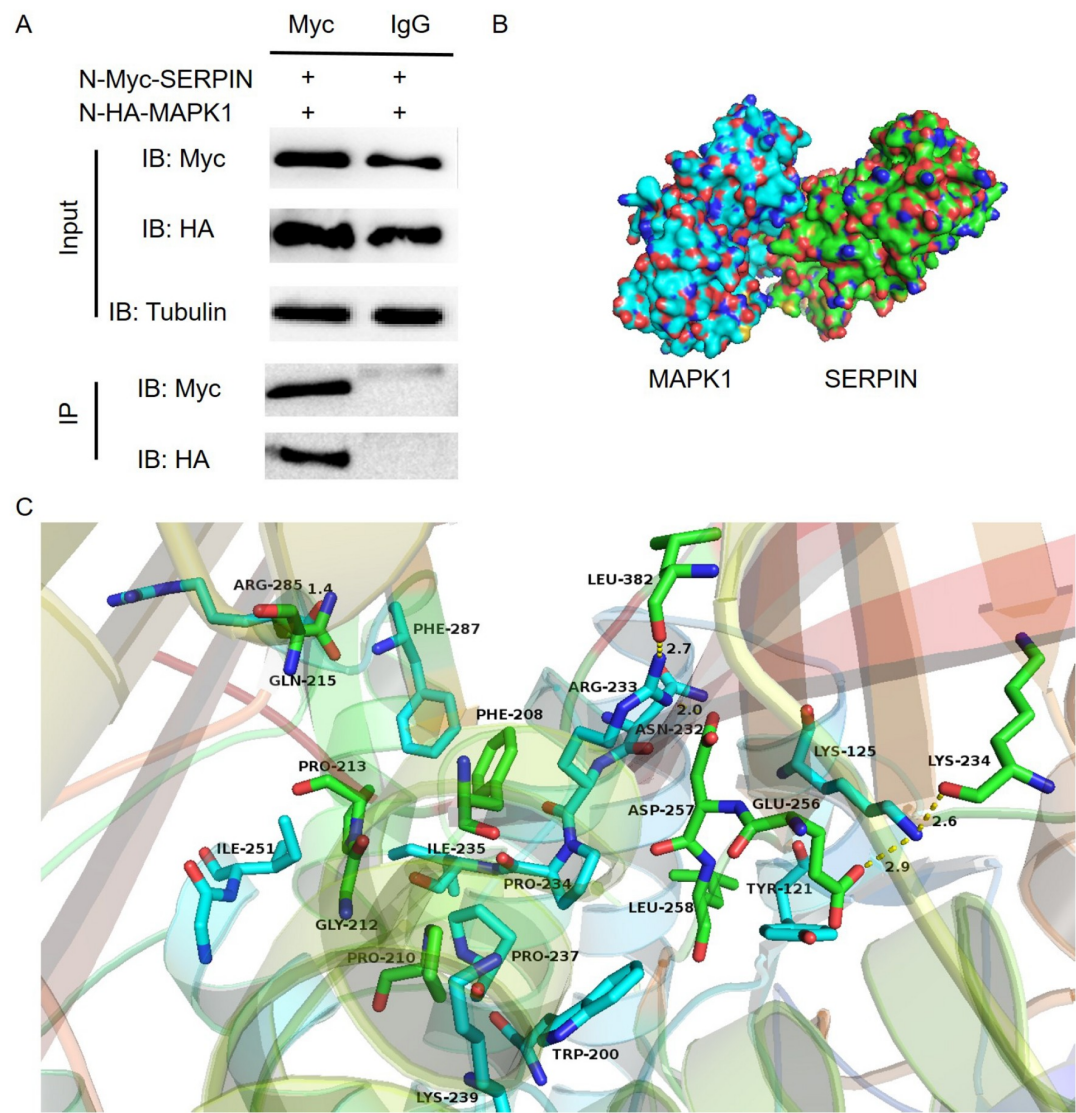
Figure 2 Predicted binding motifs of the SERPIN-MAPK1 protein interaction (A) Immunoprecipitation was performed using anti-Myc antibodies and detected by western blot analysis using anti-HA, anti-Myc, and anti-Tubulin antibodies. (B) Surface representation of the complex. Molecular docking of the SERPIN (green) and MAPK1 (blue) binding modes was performed using the ZDOCK server. (C) The detailed cartoon and stick representation of the interaction between SERPIN (green) and MAPK1 (blue), respectively.

The NCBI protein database ( http://www.ncbi.nlm.nih.gov/protein/) was used to search for the amino acid sequence of the Ea-SERPIN protein. The BLAST server ( http://blast.ncbi.nlm.nih.gov) was used to search for the template for the protein. We applied a serine protease inhibitor (PDB ID: 4p0f A) as the template for the Ea-SERPIN protein (sequence identity: 31.49%) and mitogen-activated protein kinase 3 (PDB ID: 4qtb. 1. A) as the template for the MAPK1 protein (sequence identity: 87.90%). Homology models of SERPI and MAPK1 were constructed using SWISS-MODEL ( https://swissmodel.expasy.org/). Docking studies were performed to investigate the binding mode between MAPK1 (28‒412 aa) and Ea-SERPIN (20‒363 aa) using the ZDOCK server ( http://zdock.umassmed.edu/). For docking, the default parameters were used as described in the ZDOCK server. The top-ranked pose, as judged by the docking score, was subjected to visual analysis using PyMOL 1.7.6 software ( http://www.pymol.org/). The interaction between SERPIN (green) and MAPK1 (blue) is shown in Figure 2B. One hydrophobic interaction was observed between residues Ile-251 and Trp-200 of MAPK1 and between residues Pro-213 and Leu-258 of SERPIN, resulting in strong hydrophobic binding ( Figure 2C). Detailed analysis revealed that residue Phe-287 of MAPK1 formed π-π interactions with residue Phe-208 of SERPIN. The residue Pro-237 of MAPK1 formed a CH-π interaction with the residue Pro-210 of SERPIN. The Tyr-121 residue of MAPK1 formed anionic-π interactions with the Glu-256 residue of SERPIN. Importantly, four hydrogen bond interactions were observed between Arg-233 of MAPK1 and Leu-382 and Asp-257 of SERPIN (bond lengths of 2.7 Å and 2.0 Å), Lys-125 of MAPK1 and Glu-256 and Lys-234 of SERPIN (bond lengths of 2.9 Å and 2.6 Å), and Arg-285 of MAPK1 and Gln-215 of SERPIN (bond length of 1.4 Å), which were the main binding affinities between MAPK1 and SERPIN. All these interactions helped MAPK1 anchor to the binding site of SERPIN. The above molecular simulations provide a rational explanation for the interaction between MAPK1 and SERPIN, which provides valuable information for further study of binding sites between MAPK1 and SERPIN.

After *E*. *acervulina* infection, the cells were collected at 0.5, 2, 6, 12, 24, and 36 h, and the level of MAPK1 mRNA was detected. The results showed that the level of MAPK1 mRNA increased within 6 h after *E*. *acervulina* infection, and the level decreased within 6‒12 h ( Figure 3A). These results indicated that MAPK1 may have an effect on *E*. *acervulina* proliferation in DF-1 cells. Then *MAPK1* was knocked down using a hairpin siRNA expression vector, after which the number of *E*. *acervulina* sporozoites in the DF-1 cells was counted 12 h after infection. The results showed that the number of sporozoites per 100 cells in the si-MAPK1 group was significantly lower than that in the NC group ( Figure 3B). These results indicated that MAPK1 may play an important role in *E*. *acervulina* proliferation. Furthermore, the level of *MAPK1* mRNA was significantly decreased after *MAPK1* knockdown ( Figure 3C). MAPK1 may be involved in host resistance to *E*. *acervulina* infection.

**Figure 3:**
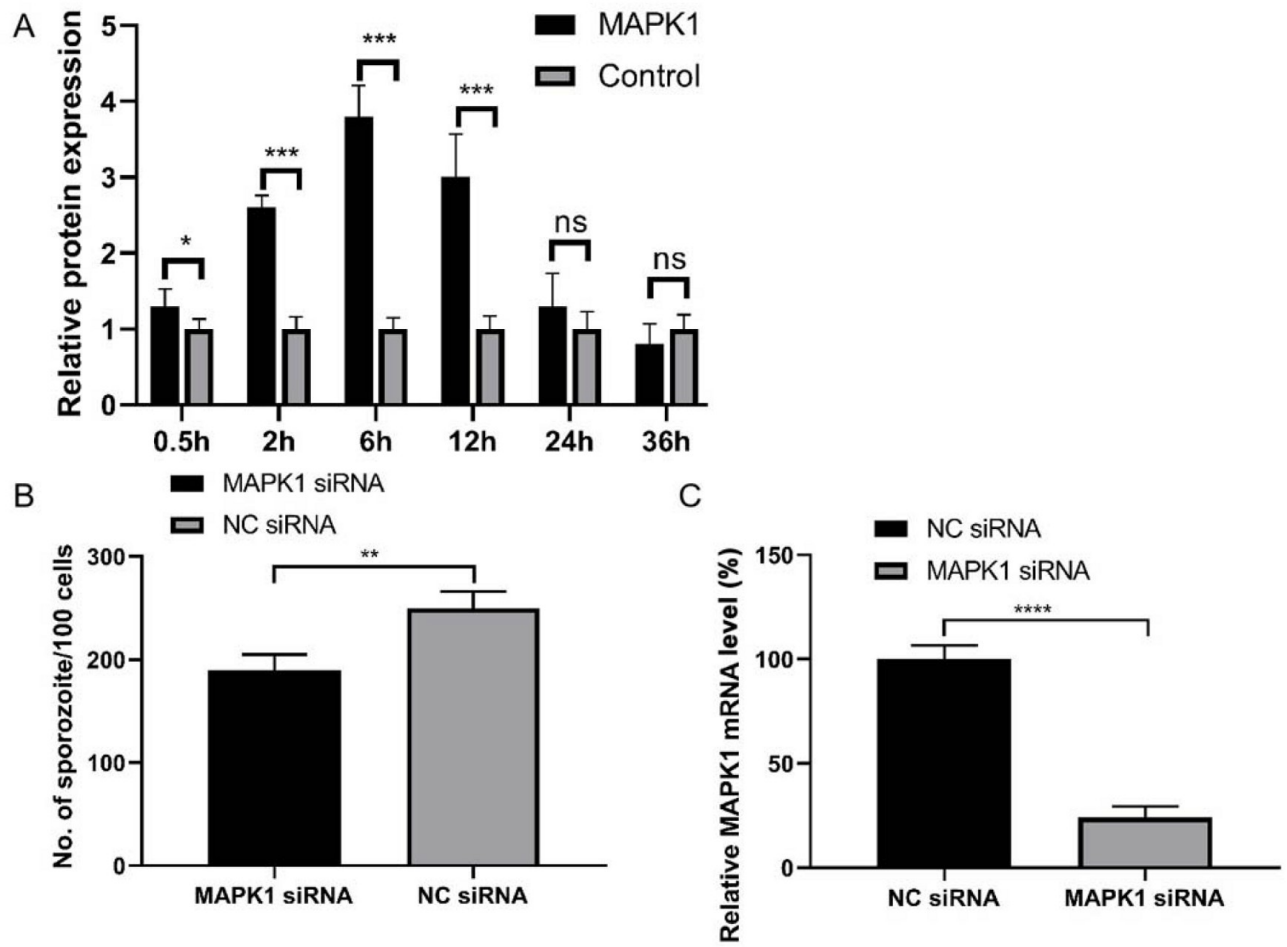
Figure 3 Effect of si-MAPK1 on *E*. *acervulina* proliferation (A) The MAPK1 level was detected after E. acervulina sporozoite infection at 0.5, 2, 6, 12, 24, and 36 h. (B) The number of sporozoites per 100 cells was counted after MAPK1 gene knockdown. (C) The MAPK1 gene was knocked down for 72 h using a hairpin siRNA expression vector, and the MAPK1 mRNA level was detected. *P<0.05, **P<0.01, ***P<0.001, ****P<0.0001.

In summary, here we first screened the SERPI-interacting protein MAPK1 using a yeast cDNA library and confirmed that SERPI interacts with MAPK1 *in vitro*. Furthermore, the interaction sites of SERPI and MAPK1 were predicted. The level of MAPK1 increased in the early stage of *E*. *acervulina* infection, but decreased over time. A decrease in MAPK transcription can significantly inhibit the proliferation of *E*. *acervulina* in cells. MAPK1 may play an important role during *E*. *acervulina* infection. Taken together, the interaction between SERPI and MAPK1 may help to elucidate the cellular functions of EaSERPI during *E*. *acervulina* infection.

## Supplementary Data

Supplementary data is available at *Acta Biochimica et Biophysica Sinica* online.
